# Lipid Metabolism and Resistance to Anticancer Treatment

**DOI:** 10.3390/biology9120474

**Published:** 2020-12-16

**Authors:** Nicolas Germain, Mélanie Dhayer, Marie Boileau, Quentin Fovez, Jerome Kluza, Philippe Marchetti

**Affiliations:** 1 UMR 9020–UMR-S 1277–Canther–Cancer Heterogeneity, Plasticity and Resistance to Therapies, Institut de Recherche contre le Cancer de Lille, University Lille, CNRS, Inserm, CHU Lille, F-59000 Lille, France; melanie.dhayer@inserm.fr (M.D.); marie.boileau.dermato@gmail.com (M.B.); quentin.fovez@inserm.fr (Q.F.); jerome.kluza@inserm.fr (J.K.); 2 Banque de Tissus, Centre de biologie-pathologie, CHU Lille, F-59000 Lille, France; 3 Service de Dermatologie, Hopital Claude Huriez, CHU Lille, F-59000 Lille, France

**Keywords:** lipid metabolism, cancer drug resistance, synthetic lethality, antimetabolic cooperativity

## Abstract

**Simple Summary:**

Cancer cells directly control nutrient uptake and utilization in a different manner from that of normal cells. These metabolic changes drive growth, proliferation of cancer cells as well as their ability to develop resistance to traditional therapies. We review published studies with pre-clinical models, showing the essential roles of lipid metabolism in anticancer drug resistance. We also discuss how changes in cellular lipid metabolism contribute to the acquisition of drug resistance and the new therapeutic opportunities to target lipid metabolism for treating drug resistant cancers.

**Abstract:**

Metabolic reprogramming is crucial to respond to cancer cell requirements during tumor development. In the last decade, metabolic alterations have been shown to modulate cancer cells’ sensitivity to chemotherapeutic agents including conventional and targeted therapies. Recently, it became apparent that changes in lipid metabolism represent important mediators of resistance to anticancer agents. In this review, we highlight changes in lipid metabolism associated with therapy resistance, their significance and how dysregulated lipid metabolism could be exploited to overcome anticancer drug resistance.

## 1. Introduction

Many oncogenic mutations resulting in the aberrant activation of several signaling pathways can reprogram cancer cell metabolism to such an extent that metabolic reprogramming is considered one of the major hallmarks of cancer [1]. Cancer cells need to reprogram their metabolism to produce enough ATP and intermediates for macromolecular biosynthesis, to meet requirements of intense cell proliferation. Changes in cellular metabolism not only result in tumor progression, but also contribute to aggressiveness features such as invasion, metastasis and cancer cell resistance to treatments [2,3,4]. In addition, cancer cells can use different metabolic programs depending on the environment. This plasticity allows cancer cells to thrive in a harsh environment, which includes oxidative stress environment or drug exposure. Interestingly, metabolic adaptation can be seen as a mean for cancer cells to survive in the presence of anticancer drugs, before a regrowth due to the acquisition of new mutations. Targeting cancer cell metabolism can be seen as a novel strategy to improve anticancer therapy and/or re-sensitize cells to anti-cancer drugs, regardless of metabolic changes participating to the acquisition of drug resistance. Results from numerous experimental studies unveiled that glucose and glutamine are the two most important nutrients expended by cancer cells, including those resistant to anticancer drugs [5,6]. Specifically, many resistant cells dysregulate the expression of metabolic genes leading to increased glucose and/or glutamine uptake followed by mitochondrial oxidation.

**Figure 1:**
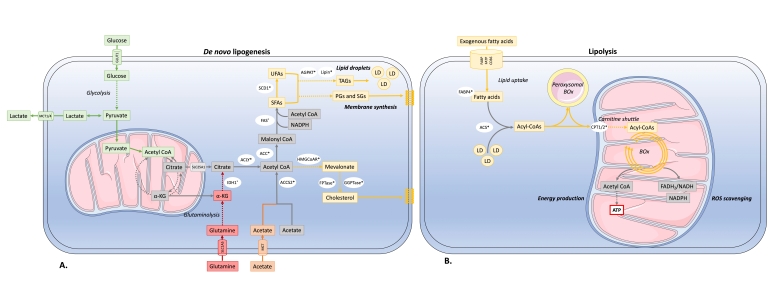
Simplified diagram of changes in lipid metabolism of cancer cells (see text for details) (**A**) De novo lipogenesis Glucose- and glutamine-derived citrate, which results from the increased glycolysis and glutaminolysis, is first converted to acetyl-coenzyme A (acetyl-CoA) by ATP-citrate lyase (ACLY). Acetyl-CoA can also be derived from acetate. Acetyl-CoA is then carboxylated to malonyl-CoA by acetyl-CoA carboxylase (ACC) and condensed by fatty acid (FA) synthase (FAS) in a repeat reaction to generate saturated FAs (SFAs) then desaturated by stearoyl-CoA desaturases (SCD1) into unsaturated FAs (UFAs). Synthesis of glycerolipids from long-chain FAs involved the enzymes 1-acylglycerol-3-phosphate acyltransferases (AGPAT) and lipin which are deregulated in resistant cancers. These lipids can be used for storage in lipid droplets, membrane biosynthesis, and signaling processes. Alternatively, acetyl-CoA can enter the mevalonate pathway to generate cholesterol. Key regulatory proteins dysregulated in resistant cancers are HMG-CoA reductase (HMG-CoAR), farnesyl prenyl transferase (FPTase) and geranylgeranyl prenyl transferase (GGPTase) (**B**) Lipolysis. FAs generated through the metabolism of triglycerides from lipid droplets (de novo synthesis) and from exogenous uptake constitute the pool of intracellular FAs that undergo fatty acid oxidation (FAO). Activation of FAs into acyl-CoA is catalyzed by Acyl coenzyme A (CoA) synthetase (ACS), then converted into FA carnitines by carnitine palmitoyl transferase 1 (CPT1) and broken down by mitochondrial β-oxidation (Box). Alternatively, peroxisomal β-oxidation catalyze the chain shortening of Acyl-CoA. FAO generates ATP production and participates to ROS scavenging through NADPH-producing reactions. Dysregulated enzymes altered in treatment-resistant cancer are indicated with an ^*^ (see text for details).

In addition to glucose and glutamine metabolism, evidence shows that lipid metabolism is also dysregulated in cancer [7]. For many years, studies have reported an aberrant accumulation of lipid droplets (LDs) in several types of cancer such as breast and lung cancer, which were correlated with higher tumor grades (for review [8]). The dysregulation of lipid metabolism in cancer is based on several complex processes contributing to tumor aggressiveness (for review [7]). Firstly, cancer cells undergo overactivation of de novo lipid synthesis or lipogenesis (Figure 1A). De novo lipid synthesis is one of the major features of cancer cells, which was observed more than 50 years ago [8]. Unlike most non-transformed cells that uptake fatty acid from exogenous dietary sources; de novo fatty acid synthesis is the major source of cancer lipids, suggesting that its inhibition might yield an acceptable therapeutic index. Lipogenesis requires the presence of acetyl-CoA, which is mainly derived from the glucose-derived pyruvate into the Krebs cycle (Figure 1). In cancer, the Warburg effect can therefore explain part of the overproduction of fatty acids [9]. Although glucose supplies carbon units for lipogenesis, alternative carbon sources can be used in case of glycolytic pathway shortage. Under hypoxia, glutamine can replace glucose to produce α-ketoglutarate (αKG) the latter will undergo reductive carboxylation via isocitrate dehydrogenase 1 (IDH1) to produce citrate and thus can significantly contribute to lipid biosynthesis in cancer cells [10]. It is noteworthy that the somatic IDH1 R132H mutation that produces the onco-metabolite 2-hydroxyglutarate increases both de novo lipogenesis and FA oxidation in acute myeloid leukemia cells [11]. Alternatively, acetate is also a carbon source for lipid metabolism in reduced nutrient conditions. Acetate metabolism provides a parallel pathway for acetyl-coenzyme A (CoA) production for lipogenesis, independent of citrate conversion to acetyl-CoA. The uptake of exogenous acetate by cancer cells depends on members of the monocarboxylate transporter family, and then acetate is converted into acetyl-CoA via acetyl-CoA synthetases (ACSS) [10,12,13,14]. Human breast cancers overexpress ACSS2, and are thus critically dependent on acetate for lipid synthesis [12]. These observations underline the intricate relationship between glycolysis, glutaminolysis, acetate metabolism and lipogenesis in cancer. High level of de novo FA synthesis results in (*i*) overproduction of neutral lipids such as triacylglycerols stored in LD that accumulate in cancer to provide a reserve of energy; (*ii*) production of phospholipids is used to build cancer cell membranes to satisfy the increased demand for cancer proliferation. Moreover, phospholipids also act as lipid messengers and intracellular signaling molecules in cancer (for review [15]). Among lipogenic enzymes, ATP citrate lyase (ACLY), the rate-limiting enzymes acetyl-CoA carboxylase (ACC) and fatty acid synthase (FAS), are the most expressed enzymes in many cancer types [16]. Particularly, FAS was identified as the lipogenesis key enzyme, and its upregulation has been correlated with a bad prognosis in many types of cancer [16,17]. Human cancers, including breast, colon and prostate cancer, have high expression and activation levels of fatty FAS, increasing the triacylglyceride (TG) synthesis stored LDs. Thus, FAS targeting in cancer is of growing interest [18]. Orlistat, a drug used for obesity treatment, was shown to target FAS by inhibiting the FAS thioesterase function leading to an antitumoral activity [19]. Besides, upregulation of the mevalonate biosynthesis pathway has been observed in many cancer types. This leads to cholesterol overproduction coming from the conversion of acetyl-CoA via the 3-hydroxy-3-methylglutaryl-CoA (HMG-CoA) reductase. Cholesterol content in cancer cell membranes and cholesterol rates were found to be aberrantly high in prostate cancer, leading to promoted cancer growth [20]. Many enzymes within the fatty-acid and cholesterol-biosynthesis pathways are upregulated in cancer by the sterol regulatory element-binding proteins (SREBP) transcription factors activated by the oncogenic PI3K/Akt/mTORC1 signaling pathway or cell cycle regulators [21].

Secondly, apart from lipogenesis, it was observed that FAs, either from extracellular sources or mobilized from internal lipid stores, can be oxidized in cancer cell mitochondria (Figure 1B). Under these conditions, lipids are used as catalytic fuels, a process called fatty acid oxidation (FAO) or lipolysis, to provide energy for cancer cells via ATP production. In some cancers, not dependent on glycolysis like B cell lymphoma, mitochondrial FAO represents the predominant pathway for energy production [22]. There are three main protein families involved in fatty acid cellular uptake: the fatty acid translocase (FAT/CD 36/SR-B2) family, the membrane fatty acid binding protein (FABPm) family and the fatty acid transport protein family (FATP) [23,24]. CD 36 overexpression was observed in colon, ovarian and breast cancers [25]. CD 36 seems to play a critical role in prostate cancer aggressiveness; its upregulation has been associated with epithelial-mesenchymal transition in hepatocellular carcinoma and was conversely correlated with leukemia survival [26,27,28]. Once within the cytoplasm, FAs are bound to fatty acid binding proteins (FABPs) then converted into acyl-CoA. Transport of acyl-CoA into the mitochondria involves the carnitine palmitoyltransferase 1 (CPT1), a protein often upregulated in cancer cells by metabolic stress [29]. Carnitine palmitoyltransferase 1 (CPT1) is the central rate-limiting enzyme of FAO. FAO takes place in the mitochondrial matrix and consists in a cyclical catabolic reaction providing nicotinamide adenine dinucleotide (NADH), flavin adenine dinucleotide (FADH2), NADPH and ATP. Interestingly, besides mitochondria, FAO was also observed in peroxisomes within cancer cells [30].

Furthermore, lipolysis and lipogenesis may coexist in cancer cells [31]. Lipid metabolism reprogramming in cancer is under the influence of the cellular environment and in particular the presence of adipocytes. Cancer-associated adipocytes represent a prominent source of external lipids for cancer cells. Studies reported a metabolic crosstalk between adipocytes and cancer cells resulting in a more aggressive phenotype of cancer cells [25,32,33,34]. Cancer cells, via the release of soluble factors such as hormone-sensitive lipase and growth differentiation factor 15 (GDF15), promote the release of FAs from neighboring adipocytes [35]. Coculture of cancer cells with adipocytes results in upregulation of the CD36 expression, leading to increased FA uptake by cancer cells. Consequently, cancer cells uptake FAs released by adipocytes, which in turn are oxidized into mitochondria and provide the energetic needs for cancer cell proliferation, survival, invasion, metastasis and drug resistance [25,31,34,36,37]. These results highlight the cross-talk between adipose tissue and cancer cells enhancing cancer FAO and aggressiveness.

Emerging evidence also suggest that dysregulated lipid metabolism could play a role in resistance to anticancer drugs. Furthermore, the dependence of cancer cells on aberrant lipid metabolism could point to lipid metabolism being a potential source of new attractive targets to eradicate cancer cells. This review highlights the role and mechanisms of lipid metabolism reprogramming induced by anticancer drugs during the development of chemoresistance in cancer cells. In addition, we discuss the potential of reversing chemoresistance via lipid metabolism regulation.

## 2. Changes in Lipid Metabolism Are Associated with Anti-Cancer Drug Resistance

It is now well-established that lipid metabolism changes are associated with resistance to conventional chemotherapies and targeted therapies in several cancers. Lipid metabolic reprogramming of resistant cancer cells includes both changes in de novo lipogenic synthesis and/or lipolytic pathway. With some exceptions [38], cancer cell resistance appears to be related to the upregulation of lipogenic or lipolytic enzyme expression. Table 1 lists evidence from studies linking lipid metabolism to drug resistance in cancer, underscoring the heterogeneity of changes observed. Firstly, changes in lipid metabolism of resistant cells is, to some extent, treatment-specific (Table 1). Resistance to tyrosine kinase inhibitors (TKIs) is associated with upregulation of de novo lipogenesis. LD accumulation, resulting from upregulation of lipogenesis, is higher in epidermal growth-factor (EGF)/TKI resistant cell lines, with aberrant activation of EGF receptor (EGFR) signaling pathway, than in cell lines with sensitive EGFR mutations [39]. This is consistent with the crucial role of the receptor tyrosine kinase signaling pathway sustaining upregulation of sterol regulatory element-binding protein (SREBP)-driven de novo lipogenesis (for review [40]). EGFR-TKI-resistant non-small-cell lung carcinoma (NSCLC) cell lines are characterized by an accumulation of LD and overexpression of Stearoyl-CoA Desaturase 1 (SCD-1), a key enzyme converting saturated fatty acids into unsaturated fatty acids [41]. Conversely, resistance to mitogen-activated protein kinase (MAPK) pathway inhibitors has generally been associated with increased FAO in BRAF mutated melanoma cells [42].

Secondly, changes in the lipid metabolism of resistant cells vary depending on the environment and cellular context. Thus, radiation resistance was associated with a significant increase in CPT1A-dependent lipolysis in nasopharyngeal carcinoma whereas radiation resistance was linked to de novo lipogenesis in head and neck squamous carcinoma [50,51]. Mechanistically, the high level of PGC-characterizing aggressive nasopharyngeal carcinoma, and not squamous cell carcinoma, can explain the difference in lipid metabolism. Indeed, PGC-1α binds to the transcription factor CCAAT/enhancer binding protein β (CEBPB) to stimulate CPT1A transcription, resulting in FAO activation [50] Moreover, the cellular environment can also contribute to lipid metabolism changes associated with treatment resistance in cancer. As discussed above, the presence of intratumoral and/or peritumoral adipocytes reduces anti-cancer drug sensitivity, partly through changes in the lipid metabolism of cancer. For example, in co-culture with adipocytes, AML blasts shift their metabolism toward FAO, a phenotype associated with chemotherapeutic resistance [62].

Overall, these data indicate that changes in lipid metabolism of resistant cells are treatment specific but also environmental and cellular context-dependent resulting in a high heterogeneity of lipid metabolisms.

## 3. Changes in Lipid Metabolism Occur in Drug-Tolerant Cancer Cells

Of note, it is interesting to stress out that lipid metabolism changes, de novo lipogenesis or lipolysis, can be unveiled upon anticancer drug exposure. Comparison of paired NSCLC tumor tissues from patients before and after Gefitinib treatment revealed a significant increase in lipid droplet content and in SCD1 expression [39]. Upon 5-fluorouracil and oxaliplatin exposure, LDs accumulate in colon cancer [63]. Acute myeloid leukemia cells treated with AraC exhibit changes in lipid metabolism as evidenced by the upregulation of CD36 expression and increased mitochondrial FAO [52]. These lipid metabolism changes are observed in drug-tolerant cells that persist in the presence of the incriminated drug and are prone to acquiring resistance via the accumulation of mutations. This observation is in agreement with the fact that lipid metabolism has been linked to the acquired anticancer drug resistance as mentioned in the previous chapter. Lipid metabolism changes in drug-tolerant cells can correspond to two different processes.

Firstly, several studies suggested that aberrant lipid metabolism could be seen as metabolic shift allowing cancer cells to adapt to treatment-induced cellular stress. Almost all anticancer drugs including conventional chemotherapies and targeted therapies induce cancer cell stress that can ultimately lead to cell death. It was reported that several cellular stressors like lack of nutrients, high levels of reactive oxygen species (ROS) promote de novo lipogenesis [64]. Thus, lipid metabolism changes can represent an active and protective response to stress, mediated by cancer drugs promoting survival even in the presence of the drug. Survival mechanisms are highly variable depending on the nature of drug-induced stress (see next chapter below). One example of lipid metabolism adaptive changes under drug exposure is the BRAF mutated melanoma tolerant to MAPK inhibitors. MAPK inhibitors, such as BRAF inhibitors and MEK inhibitors, which are currently used in combination, demonstrated their high efficacy to treat BRAF-mutant melanoma, however an acquired resistance undoubtedly develops [65]. The exposure to MAPK inhibitors strongly decreases glucose uptake and glycolysis, which in turn leads to ER stress-induced apoptosis [66]. To survive to the metabolic stress induced by MAPK inhibitors, tolerant BRAF-mutated melanoma must become dependent on oxidative respiration through the use of glutamine [67]. Recently, evidence indicated that tolerant BRAF-mutated cells also use lipids as additional nutrient sources to survive in the presence of MAPK inhibitors [30]. Thus, tolerant cells switch from glycolysis to complete FAO both in peroxisomes and mitochondria to adapt to metabolic stress induced by MAPK inhibitors. The PPARα/PGC-1α transcriptional axis is directly involved in the activation of FAO in persistent cells through the upregulation of two limiting FAO-related genes, the peroxisomal ACOX1 and the mitochondrial CPT1, suggesting a cooperation of these two organelles for the survival of persistent cells. Another example of metabolic switch from glycolysis toward lipid metabolism upon anticancer therapy is represented by the tumor response to antiangiogenic drugs [61]. In preclinical animal models, antiangiogenic drugs such as anti-VEGF induce hypoxia, which in turn reprograms lipid metabolism of colorectal cancer and pancreatic ductal adenocarcinoma implanted in adipose tissues. In response to antiangiogenic drugs, cancer cells display a significant increase in external FA uptake and transportation through the upregulation of FA transporters (CD36, FABP, SLC27) as well as an augmentation of mitochondrial CPT1-mediated FAO. Lipid reprogramming allows cancer cells to survive and grow in the presence of antiangiogenic drugs. Lipid reprogramming would represent a compensatory mechanism to the lack of glucose supply induced by antiangiogenic drug-mediated vessel reduction.

Secondly, apart from an adaptive process, cancer cell exposure to anticancer drugs can result in an enrichment of a pre-existing cellular subpopulation characterized by aberrant lipid metabolism. According to this hypothesis, lipid metabolism is a metabolic state allowing a sub-population of cancer cells to escape the effects of anticancer drugs when other cancer cells die. This subpopulation could be represented by cancer stem cells (CSCs), a highly resistant subset of cancer cells. Indeed, it is well established that even if a successful cancer therapy abolishes the bulk of tumor cells, CSCs can survive to standard cancer treatments and are at the core of clinical relapse [68]. Interestingly, lipid metabolism of CSCs differs from that of differentiated cancer cells [69,70,71]. Changes in lipid metabolism of CSCs are heterogeneous including an increase in de novo lipogenesis or in lipid uptake and fatty acid oxidation depending on the cancer cell type (for review [70]). Similarly, a subset of leukemic stem cells (LSCs), residing in adipose tissue and expressing the fatty acid transporter CD36, displays a high level of FAO activity responsible for a singular drug resistance profile [72]. Thus, lipid metabolism could characterize a pre-existing subpopulation of resistant CSCs selected by anticancer drugs.

## 4. Changes in Lipid Metabolism Contribute to Anticancer Drug Resistance

In many preclinical models, lipid metabolism inhibition can reverse the resistance of cancer cells to cancer drugs suggesting that lipid metabolism may play a role in drug resistance. The question is how can lipid metabolic reprogramming contribute to anticancer drug resistance?

### 4.1. Lipid Metabolism Protects Cancer Cells from Stress Induced by Anticancer Drugs

#### 4.1.1. Lipid Metabolism Counteracts Oxidative Stress Induced by Anticancer Drugs

The oxidative stress induced by anticancer drugs result in peroxidation of lipid membranes, oxidative modifications of proteins and DNA. Doxorubicin, which possesses an anthracycline skeleton, generates ROS leading to DNA damage followed by anticancer activity. Likewise, vinca alkaloids increase intracellular ROS production by depleting the intracellular GSH causing DNA damages. Changes in lipid metabolism elicit a cytoprotective response to oxidative stress in several different ways: (i) lipid droplets decrease ROS toxicity thereby increasing cancer cell survival [73] Production of LDs counteracts membrane lipid peroxidation. Thus, LDs reorganize the oxidized lipids resulting in a reduced percentage of oxidized lipids in cell membranes [64]. It is also plausible that LDs may sequester harmful molecules such as ROS or lipid peroxides protecting cells from oxidative stress [74]; (ii) moreover, the degree of lipid saturation influences the physicochemical properties of cell membranes. Increased saturated FA levels make cell membranes more resistant to ROS-dependent peroxidation and to cell death by ferroptosis [75]. Activation of de novo lipogenesis generates palmitate that is converted into monounsaturated fatty acids by SCD-1. Although SCD-1 expression is frequently upregulated in cancer, this is an oxygen-dependent enzyme, which is inactivated under hypoxic conditions, a situation frequently observed in cancer [76]. Thus, one might assume that increased palmitate de novo biosynthesis uncoupled to desaturation, observed under hypoxia, can result in resistance to oxidative stress [75]; (iii) fatty acid oxidation has been associated with resistance to oxidative stress induced by chemotherapeutic agents or radiation [77]. It was reported that a crucial function of FAO is generating the reducing equivalent NADPH to maintain antioxidant balance. Under stress conditions, cancer cells sustain NADPH levels by increasing FAO and the concomitant downregulation of fatty acid de novo biosynthesis [78]. Pharmacological inhibition of FAO with the CPT-1 inhibitor, etomoxir, diminishes NADPH levels and glutathione content leading to an elevation of intracellular ROS [79]. Thus, FAO could be as important as fatty acid biosynthesis for cancer cell redox homeostasis and protection against drug-mediated oxidative stress.

#### 4.1.2. Lipid Metabolism Counteracts ER Stress Induced by Anticancer Drugs

Lipid droplet accumulation was evidenced to support colorectal cancer resistance to 5-fluorouracil (5-Fu) and oxaliplatin by inhibiting ER stress [63]. Indeed, the lysophosphatidylcholine acyltransferase 2 (LPCAT2), a lipid droplet-localized enzyme, promotes phosphatidylcholine synthesis and thereby attenuates drug-promoted ER stress, and finally blocks caspase activation and subsequent apoptosis [63] In clear-cell renal cell carcinoma, the constitutive activation of hypoxia-inducible factor-2α (HIF2α) promotes expression of the lipid droplet coat protein PLIN2 that contributes to the abundance of intracellular LDs. It is to note that HIF2α/PLIN2–mediated lipid storage is protective against pharmacologic ER stress reinforcing the potential role of lipid metabolism in ER stress resistance induced by anticancer drugs [80].

#### 4.1.3. Lipid Metabolism Reduces Genotoxicity Induced by Anticancer Drugs

Overexpression of FAS, the key enzyme of de novo lipogenesis pathway, triggers cancer resistance to genotoxic drugs by increasing DNA repair [81]. Mechanistically, FAS transcriptionally upregulates PARP-1 expression via concomitant NF-κB inhibition and SP1 activation. Consequently, PARP-1 mediates NHEJ repair and resistance to drug-induced DNA damages [81].

#### 4.1.4. Lipid Metabolism Reduces Metabolic Stress Induced by Anticancer Drugs

Targeted therapies such as MAPK inhibitors inhibit glucose uptake and antiglycolytic effects triggering energy stress conditions contributing to the promotion of cancer cell death (see above). Acetyl-CoA derived from fatty acid oxidation can fuel the mitochondrial TCA cycle to reduce energetic stress. This metabolic shift towards FAO, is often orchestrated by the AMP-dependent protein kinase (AMPK). Activation of AMPK in response to low energy levels boosts energy production through a mitochondrial FAO increase [82] and thereby enables cancer cell survival in the presence of anticancer drugs.

### 4.2. Lipid Metabolism Inhibits Drug-Induced Cancer Cell Death

Lipid metabolism can also interfere with the process of apoptotic cell death induced by anticancer drugs through two distinct mechanisms: (i) LDs were shown to remove apoptosis-related proteins, such as BCl-2 family members, from mitochondria by direct contact between outer mitochondrial membrane and lipid droplet surface [83]. This process prevents cytochrome c release and could inhibit drug-induced apoptosis; (ii) Mitochondrial lipid composition influences drug-mediated apoptosis through mitophagy regulation [84] In stress conditions, increased percentage of sphingosine-1-phosphate molecules in mitochondrial membranes activates a protective mitophagy to impede the drug-induced apoptosis [84].

### 4.3. Lipid Metabolism Contributes to the Maintenance of Drug-Resistant Cancer Stem Cells

A large body of evidence indicates that human cancers emerge from CSCs, which are intrinsically resistant to many anticancer treatments including conventional chemotherapies, targeted drugs and radiation. CSCs are also the main source of cancer relapse. Interestingly, lipid metabolic reprogramming contributes to CSCs expansion and survival therefore enhancing the occurrence of chemoresistance [85]. Several lipogenic genes are reprogrammed in CSCs and are critical for CSCs maintenance. Although these mechanisms require further investigation, FAS was found to be involved in CSCs survival in several cancer cell types such as glioma, pancreatic tumors or breast cancer [86]. Knockdown of ACLY inhibits epithelial–mesenchymal transition, a phenomenon related to cancer stemness [87]. Likewise, pharmacological inhibition of ACC with soraphen A, significantly reduces ALDEFLUOR^+^ CSC content in the MCF7 breast cancer cell line [88]. Activation of SCD1 and the subsequent production of mono-unsaturated fatty acids further activates stemness program through Wnt signaling in lung NSCLC [69] conversely the inhibition of desaturases reduces cancer stemness markers [70]. HMG-CoA reductase also stimulates stemness via several signaling pathways including the mevalonate, Rho GTPases and YAP/TAZ pathways in brain CSCs [33,41,89,90,91,92,93,94,95,96]. All of these elements evidence that CSC survival relies on lipid synthesis. Additionally, several reports indicate that FAO could also be critical for maintaining the CSCs pool. Inhibiting FAO using the CPT-1 inhibitor, etomoxir reduces the number of leukemic progenitors and stem cells [97]. Similarly, pharmacological inhibition of FAO by etomoxir constraints the enlargement of liver CSCs and sensitizes CSCs to the tyrosine kinase inhibitor treatment, sorafenib [98].

Overall, these observations identify the rewiring of lipid metabolism as a novel and important mechanism of adaptive resistance to anticancer drugs.

## 5. Lipid Metabolism a Source of Potential Therapeutic Targets to Kill Resistant Cancer Cells?

As detailed above, recent studies have shown that cancer cells develop changes in lipid metabolism, which is different from that of non-proliferative differentiated cells. These observations open up new avenues for the exploitation of lipid metabolism as a source of new therapeutic targets. Natural and synthetic agents that affect lipid metabolism in cancer is a rapidly growing field that was recently reviewed elsewhere [40]. The focus of our discussion is (i) on the interest of combining lipid-targeted drugs with current anticancer therapies and (ii) on the possibility of reversing cancer cell resistance.

Numerous pharmacological inhibitors have been developed for almost all enzymes of lipid metabolism and some compounds are used in clinical trials in association with conventional therapies (Table 2). Combination treatments bear several advantages: (i) In most cases the use of lipid targeted drugs alone is cytostatic, thus blocking cell proliferation or the spread of metastasis but insufficient to kill cancer cells. Conversely, combination treatment is often synergistic and exerts a real potential to eradicate cancer cells. In resistant ovarian cancer, the FAS inhibitor, orlistat, interacts synergistically with the specific Her-2 inhibitor trastuzumab leading in vitro to a significant increase in apoptosis [99]. Regarding targeting lipid catabolism, Etomoxir, one of the first CPT1 inhibitor, proved to be synergistic with many anticancer therapies [97,100,101,102,103]; Likewise, the SCD1 inhibitor, g-PPT, which reduces the synthesis of polyunsaturated fatty acids, inhibits TG synthesis and lipid droplet accumulation in cancer cells. Used in combination with gefitinib, g-PPT effectively reduces drug resistance and promotes cancer cells apoptosis [39]. However, SCD1 inhibition is potentially responsible for adverse effects since the accumulation of SCD1 substrates result in inflammation, atherosclerosis, as well as liver and pancreatic dysfunction in pre-clinical models [104] (ii) The rationale for such a combination is also based on the fact that lipid targeted drugs and standard treatment address two complementary aspects of the metabolism and that neither drug alone can succeed in promoting cell death. As previously mentioned, some anticancer drugs such as Ara-C or MAPK inhibitors are potent glycolytic inhibitors that force surviving cancer cells to use lipid metabolism, thus making them addicted to lipid metabolism. These combinations increase the therapeutic efficacy and cancer specificity of lipid metabolism targeted therapies. Thus, combination drug therapy displays antimetabolic cooperativity reducing the metabolic flexibility of cancer cells.

Besides, due to the role of lipid metabolism in the acquisition of treatment resistance, targeting lipid metabolism could be used to re-sensitize cancer cells to standard treatments. FAS inhibitors have shown in-vivo and in-vitro anticancer effects and are also responsible for re-sensitization of cancer cells to conventional therapy [59,99,105,106,107]. Thus, FAS inhibition reduces the level of HER2 expression and significantly increases the sensitivity of cancer cells to HER2 inhibition [105] Similarly, inhibition of CPT1 re-sensitizes chronic lymphocytic leukemia cells resistant to the tyrosine kinase inhibitor, ibrutinib [97] and inhibition of SCD1 reverts resistance to cisplatin in lung cancer stem cells [96].

One major interest in lipid targeting for cancer treatment is the possibility of using existing clinically-approved drugs originally developed for other lipid-related diseases. This drug repositioning in oncology has the main advantage of improving safety and reducing costs. Besides the anti-obesity drug, orlistat (see above), statins represent a classic example of drug repositioning in oncology. Statins are currently the most efficient drugs to reduce circulating cholesterol, with few side effects including muscle pain and occasionally liver inflammation, and thereby are used in preventing the development of cardiovascular diseases [125]. Molecularly, statins inhibit the rate-limiting enzyme HMG-CoA reductase and therefore block the mevalonate pathway dependent on cholesterol biosynthesis. In preclinical models, statins have shown their effectiveness in re-sensitizing multidrug resistant (MDR) colon and breast cancer cell lines to doxorubicin, mainly through the modification of the lipid membrane’s composition [114,115]. However, results from cohorts and case-control studies, as well as meta-analyses, on the efficacy of statins in reducing cancer mortality are conflicting and nonconclusive [126,127]. Other interesting examples of drug repositioning in this context are perhexiline and ranolazine, two CPT inhibitors, already commercially available in some countries for angina pectoris treatment. In a recent study, perhexiline was used in association with oxaliplatin to effectively inhibit gastric tumor progression in an in vivo pre-clinical model [123]. In this model perhexiline re-sensitized cells to oxaliplatin by bocking FAO and intensifying intracellular ROS accumulation. Perhexiline and ranolazine could be seen as an alternative to the use of the prototypic CPT inhibitor, etomoxir, which proved to be particularly hepatotoxic in clinical trials [128].

Based on preclinical data presented above, combination therapy consisting of standard anticancer therapies and lipid metabolism inhibitors would be effective for treating resistant cancers.

## 6. Conclusions

Lipid metabolism plays a central role in cancer resistance, not only via an increased availability of lipids conferred by adipocyte environment but also through profound changes in cancer cell lipid metabolism. It is particularly interesting to note that CSCs, cells that are known to be at the center of resistance mechanisms and relapse, have an increased dependence on lipid metabolism. This could offer a very large number of potential targets, as reported in this review. Changes in the lipid metabolism of cancer cells has been overlooked since conventional 2D cell culture is unable to recapitulate the tumor environment. Moreover, conventional culture medium does not recapitulate normal fatty acid environment as reported by Else [129]. Optimization of culture conditions, via 3D co-culture models, could be essential to minimize the gap between in vitro and in vivo, when it comes to studying cancer resistance to treatment [130]. The accumulating knowledge on lipid metabolism of resistant cancer cells has opened up new avenues for developing therapeutic approaches Nevertheless, the flexibility of metabolic networks constitutes challenging issues for lipid metabolism targeting in cancer therapy. Thus, associating lipid metabolism-targeted drugs to standard therapies could represent an interesting strategy for cancer treatment. More fundamental and clinical studies are warranted to validate lipid metabolism as a valuable source of targets for cancer therapy.

## Figures and Tables

**Table 1:** Non-exhaustive list of changes in lipid metabolism associated with resistance to anticancer treatments.

Cancer		Resistance to Drugs		Lipid Metabolism Reprogramming in Resistant Cells		
Type	Model	Drug	Drug Target	Pathway	Mechanism	Reference
Pancreatic adenocarcinoma	In vitro and in vivo xenografts	Gemcitabine	Thymidylate synthetase inhibition	Increased lipogenesis	Increased FASN expression	[43]
Ovarian cancer	In vitro cell lines and primary cells	Cisplatin	DNA binding	Increased lipogenesis	Increased FASN expression	[44]
Breast cancer	In vitro			Increased lipogenesis	Increased FASN expression	[45]
Bladder cancer	In vitro			Increased lipogenesis	Increased cytosolic ACSS2 expression	[46]
Ovarian cancer	In vitro and in vivo xenografts	Carboplatin	DNA binding	Increased lipolysis	Adipocyte-Induced FABP4 Expression	[47]
Breast cancer	In vitro cell lines and patient tissue and in vivo	Paclitaxel	Antimicrotubule agent	Increased lipolysis	High mRNA levels of CPT1B and FAO	[48]
Breast cancer	In vitro	Radiation therapy	DNA double strand breaks	Increased lipolysis	High CPT1A andCPT2 expression and increased FAO	[49]
Nasopharyngeal carcinoma	In vitro cell lines and tissue assay			Increased lipolysis	High CPT1A expression and increased FAO	[50]
Head and Neck Squamous Cell Cancer	In vitro			Increased lipogenesis and decreased lipolysis	Increased FASN expression	[51]
AML	In vitro primary cells and in vivo patient derived xenografts	Cytarabine	Nucleoside analogue of cytosine	Increased lipolysis	Increased CD36 expression	[52]
Acute myeloid leukemia	In vitro	Mitoxantrone	Type II topoisomerase inhibitor	Increased lipogenesis and lipolysis	Increased lipid droplets and increased OXPHOS	[53]
Breast cancer	In vitro	Doxorubicin and mitoxantrone	DNA binding and type II topoisomerase inhibitor	Increased lipogenesis	Increased FASN expression	[54]
Burkitt lymphoma	In vitro	Bortezomib	Inhibition of the 26S proteasome	Increased lipogenesis	Induction of a GGPP-dependent survival pathway	[55]
Melanoma	In vitro and in vivo xenografts	BRAFi and MEKi	Selective inhibitors of mutated BRAF/MEK	Increased lipolysis	Increased peroxisomal β-oxidation	[30]
Non-small cell lung cancer	In vitro and in vivo xenografts	Gefitinib	Inhibitor of the epidermal growth factor receptor (EGFR) tyrosine kinase	Increased lipogenesis	Increased membrane fluidity by high lipid droplet content and SCD1 expression	[41]
	In vitro and in vivo xenografts			Increased lipogenesis	High cholesterol level in lipid rafts	[56]
Breast cancer	In vitro			Increased lipogenesis	EGFR sequestrated within plasma membrane cholesterol lipid rafts	[57]
Breast cancer	In vitro and in vivo xenografts	Lapatinib	Inhibitor of EGFR/HER1 and HER2 receptors	Unknown	Increased adipocyte lipolysis	[58]
Breast cancer	In vitro	Trastuzumab	Inhibitor of HER2 receptors	Increased lipogenesis	Increased FAS promoter	[59]
Breast cancer	In vitro	Tamoxifen	Inhibitor of oestrogen receptors (ERs)	Increased lipogenesis	Increased cholesterol pathway gene expression	[60]
Multiple cancer models	In vitro and in vivo xenografts	Anti-Angiogenic drugs	Inhibitors of vascular endothelial growth factor (VEGF)	Increased lipolysis	Increased FFA uptake and FAO induced by hypoxia	[61]

**Table 2:** Non-exhaustive list of drugs targeting lipid metabolism used in association with standard treatments in resistant cancer.

Pathway/Enzyme		Lipid Targeted Drug	Specific Mechanism	Development Stage	Preclinical/Clinical Model	Drug Combination	Effects
**FA synthesis**	FAS	G28UCM		Preclinical	Breast cancer xenografts, anti-HER2 resistant cell lines	Trastuzumab, Lapatinib, Gefitinib, Erlotinib	Re-sensitizes to drugs, increases apoptosis and decreases activation of HER2 [105]
		C75/cerulenin	Inhibition of β-ketoacyl-synthase activity	Preclinical	Ovarian and breast cell lines	Trastuzumab	Increases cytotoxicity and suppression of HER2 overexpression [59]
		Orlistat	Inhibition of thioesterase domain (unspecific of FAS)	Preclinical	Chemoresistant ovarian cancer cell lines	Trastuzumab	Increases cytotoxicity and suppression of HER2 overexpression [99]
					Prostate resistant cell lines	Taxanes	Decreases viability, increases apoptosis and enhances microtubule stability [106]
					Hormone-refractory, TRAIL-resistant prostate cancer cells	TRAIL	ROS-mediated increase in apoptosis [107]
	SCD1	A939572	SCD1 inhibitor enzymatic activity	Preclinical	Lung cell lines and xenografts	Gefitinib	Reduces tumor progression and inhibits EMT phenotype of cancer cells [41]
					Clear renal cell carcinoma cell lines and xenografts	Temsirolimus	Decreases tumor cell proliferation and induction of apoptosis in vitro and in vivo [89]
		MF-438		Preclinical	Lung cancer stem cells	Cisplatin	Inhibits 3D spheroids formation, induces CSCs apoptosis [96]
**FA synthesis**		SSI-4		Preclinical	Hepatocellular carcinoma cell lines and sorafenib-resistant xenografts	Sorafenib	Suppresses liver TICs and sorafenib resistance [92]
		20(S)-protopanaxatriol (g-PPT)		Preclinical	TKI-resistant non-small cell lung cancer cell lines and xenografts	Gefitinib	Reverses resistance and inhibits activation of p-EGFR [39]
	ACS	Triacsin C	Inhibitor of acyl-CoA synthetase 1 and 4	Preclinical	Breast cancer cell lines	Paclitaxel, Doxorubicin, Cisplatin	Inhibits proliferation and reduces ABCG2 expression in cells overexpressing ACSL4 [108]
		N-(2,3-di-2-thienyl-6quinaxalinyl)-N’-(2-methoxyethyl) urea	Inhibitor of acyl-CoA synthetase 2	Preclinical	Bladder cancer cell lines resistant to Cisplatin	Cisplatin	Abrogation of resistance to cisplatin [109]
**Cholesterol synthesis**	HMG-CoA reductase	Statins	Inhibitors of 3-hydroxy-3-methylglutaryl coenzyme A (HMG-CoA) reductase	Preclinical	Various type of cancers cell lines and xenografts (AML, CLL, MDR-colon cancer, MDR-breast cancer cell lines…)	Venetoclax, Idarubicin+ Cytarabine, ATRA, Cytarabine, Daunorubicin, Doxorubicin	Growth inhibition, increase in apoptosis [110,111,112,113,114,115]
				Phase I/II/III/IV studies	Various type of cancers (Lung, breast, liver …)	See studies for details	
	FPTase	L-744,832	Selective inhibitor of FPTase	Preclinical	Mammary and salivary carcinomas xenografts	Doxorubicin	Tumor regression [116]
					Pancreatic ductal adenocarcinoma cell lines and xenografts	Ionizing radiations	Enhances the cytotoxic effect of ionizing radiation [117]
**Cholesterol synthesis**	FPTase	SCH66336 (Lonafarnib)		More than 35 phase I/II/III studies	Gliosarcoma, bladder cancer, head and neck cancer, CML, liver cancer, etc.	See studies for details	
		R115777 (Zarnestra, Tipifarnib)		More than 80 phase I/II/III studies	CML, non-small cell lung cancer, colorectal, etc.	See studies for details	
	GGPTase	GGTI-2418 (PTX-100)		1 Phase I study (NCT03900442)	Advanced malignancies	See studies for details	
		GGTI-298	Geranylgeranyl transferase 1 inhibitor	Preclinical	Non-small cell lung cancer cell lines	Gefetinib	Synergistic effect on the inhibition of proliferation, induces apoptosis and reduces migration [118]
**Esterification and storage**	AGPAT	CT-32228		Preclinical	Chronic myelogenous leukemia resistant to Imatinib	Imatinib	Inhibits proliferation, induces apoptosis [119]
	Lipin	Propanolol	Inhibition of Lipin-1	Preclinical	Prostate and breast adenocarcinomas	Rapamycin	Sensitizes to Rapamycin [120]
	LD	Pyrrolidine-2	Reversible inhibitor of cPLA2α	Preclinical	Glioblastoma cell lines	Curcumin	Enhances Curcumin effect and caspase-3 mediated cell death [121]
**Catabolism and uptake**	FAT/CD 36	Anti-CD36 antibody	Irreversible inhibition of CD36	Preclinical	Tamoxifen resistant breast cancer cells	Tamoxifen	Reduces proliferation [122]
	FABP	BMS309403	Inhibitor of FABP4	Preclinical	Carboplatin-resistant ovarian cancer cell lines and xenografts	Carboplatin	Reduces tumour burden and increases the sensitivity towards carboplatin in vitro and in vivo [47]
	CPT1/CPT2	Etomoxir	CPT1 inhibitor	Preclinical	Various type of cancers cell lines and xenografts (lung adenocarcinoma, ovarian cancer, AML, prostate …)	ABT-737, Cytosine arabinoside, Arsenic trioxide, Paclitaxel, Cisplatin, Enzalutamide	Reduces tumour growth, sensitizes to apoptosis [97,100,101,102,103]
		Perhexiline	CPT1 and 2 inhibitors	Preclinical	Gastrointestinal cancer cell lines and xenografts	Oxaliplatin	Enhances apoptosis and increases ROS in vitro, suppresses tumor progression in vivo [123]
					Breast cancer cell lines and xenografts	Lapatinib	Inhibits cell proliferation in vitro and tumor growth in vivo [124]
					Enzalutamide-resistant prostate cancer cell lines and xenografts	Enzalutamide	Inhibits cell proliferation in vitro and tumor growth in vivo [103]
		Ranolazine	CPT1 inhibitor	Preclinical	Prostate cancer	Enzalutamide	Inhibits cell proliferation in vitro and tumour growth in vivo [103]

* NCT03808558, NCT04352361, NCT02223247, NCT03938246, NCT02980029, NCT03179904, NCT03032484.
